# Food insecurity, vitamin D insufficiency and respiratory infections among Inuit children

**DOI:** 10.3402/ijch.v75.29954

**Published:** 2016-02-15

**Authors:** Sze Man Tse, Hope Weiler, Tom Kovesi

**Affiliations:** 1 Division of Pediatric Respiratory Medicine, Department of Pediatrics, Sainte-Justine University Health Center, Montréal, Quebec, Canada; 2 School of Dietetics and Human Nutrition, McGill University, Montreal, Quebec, Canada; 3 Division of Pediatric Respirology, Department of Pediatrics, Children's Hospital of Eastern Ontario, Ottawa, Quebec, Canada

**Keywords:** food security, vitamin D, Inuit, bronchiolitis, lower respiratory tract infections, crowding

## Abstract

**Background:**

Food insecurity, vitamin D deficiency and lower respiratory tract infections are highly prevalent conditions among Inuit children. However, the relationship between these conditions has not been examined in this population.

**Objective:**

The objective of this study was to examine the relationship between food insecurity and severe respiratory infections before age 2 years and health centre visits for a respiratory problem in the past year. We also explored the relationship between serum vitamin D status and respiratory outcomes in this population.

**Design:**

We included children aged 3–5 years who participated in a cross-sectional survey of the health of preschool Inuit children in Nunavut, Canada, from 2007 to 2008 (n=388). Parental reports of severe respiratory infections in the first 2 years of life and health care visits in the past 12 months were assessed through a questionnaire. Child and adult food security were assessed separately and serum 25-hydroxyvitamin D3 levels were measured in a subgroup of participants (n=279). Multivariate logistic regression was performed to assess the association between food security, vitamin D and each of the 2 respiratory outcomes.

**Results:**

Child and adult food insecurity measures were not significantly associated with adverse respiratory outcomes. Household crowding [odds ratio (OR)=1.51, 95% confidence interval (CI) 1.09–2.09, p=0.01 for the child food security model] and higher birth weight (OR=1.21, 95% CI: 1.02–1.43, p=0.03) were associated with reported severe chest infections before age 2 years while increasing age was associated with decreased odds of reported health care visits for a respiratory problem (OR=0.66, 95% CI: 0.48–0.91, p=0.02). Neither vitamin D insufficiency nor deficiency was associated with these respiratory outcomes.

**Conclusions:**

Using a large cross-sectional survey of Inuit children, we found that household crowding, but not food security or vitamin D levels, was associated with adverse respiratory outcomes. Further studies are warranted to examine the impact of decreasing household crowding on the respiratory health of these children.

Canadian Inuit children have one of the highest rates of lower respiratory tract infections (LRTI) in the world, with admissions for LRTI being up to 10 times more frequent compared with other Canadian populations (1). Studies have documented an alarming hospitalization rate of 484 per 1,000 infants under 6 months of age for bronchiolitis (2). Rates of complications are also high, with 12.8% of admitted children requiring intubation (2). Bronchiolitis and pneumonia are clinically difficult to differentiate among children with LRTI. Among Inuit children, respiratory syncytial virus (RSV) has been identified as a common cause of LRTI (3). However, the rates of pneumonia, tuberculosis and post-infectious respiratory complications such as bronchiectasis are also substantially higher than in the rest of Canada (4). Despite the introduction of a 7-valent pneumococcal conjugate vaccine (PCV7) in 2002 and the 13-valent vaccine (PCV13) in 2010 in Northern Canada, pneumococcal pneumonia remains a major issue (5). Studies examining the effect of PCV7 in Northern communities have found that while there has been a decrease in invasive pneumococcal disease caused by serotypes included in the vaccine, there was an increase in disease caused by serotypes not included in PCV7 (6,7). A number of risk factors have been associated with respiratory disease prevalence and/or severity in Inuit children, including household overcrowding, passive and in utero smoke exposure, lack of breastfeeding and reduced ventilation in the house (8,9). Of these, household overcrowding and passive smoke exposure are highly prevalent.

Food insecurity is a prevalent problem among the Inuit, with nearly 70% of Inuit preschoolers residing in food insecure households (10). According to the 1996 World Food Summit, food security is achieved when “all people, at all times, have physical and economic access to sufficient, safe, and nutritious food to meet their dietary needs and food preferences for an active and healthy life” (11). Household food insecurity has been recognized as a determinant of childhood undernutrition (12) and other adverse health outcomes (13–15), including pneumonia (16), and nutritional deficiencies (17,18). Food insecurity has also been associated with childhood tuberculosis (19); however, the relationship between food insecurity and other respiratory infections is unclear.

Vitamin D deficiency is another highly prevalent problem among Inuit, due to their limited sun exposure in the winter (20) and inadequate dietary intake (21). Vitamin D deficiency has been associated with increased risk of respiratory infections in children (22,23). One trial found that supplementation with vitamin D was associated with a reduction in the risk of acute respiratory infections in Mongolian children (24). However, the biologic mechanisms underlying these associations have not been clearly elucidated. Despite the high prevalence of vitamin D deficiency among Inuit children, it is not known whether vitamin D deficiency is associated with respiratory infections in this population.

This study examined the relationship between food insecurity and adverse respiratory outcomes among Inuit children, namely reported severe respiratory infections before age 2 years and health centre visits for a respiratory problem. In addition, we explored the relationship between serum vitamin D status and respiratory outcomes in this population.

## Methods

### Subjects

Between the late summer and fall of 2007 and 2008, a cross-sectional survey of the health of preschool Inuit children was conducted in 16 communities of Nunavut, Canada. The details of this survey have been reported previously (10). Briefly, Inuit children aged 3–5 years from the surveyed communities were eligible to participate. They were recruited from community health centres’ lists of age-appropriate children and randomly selected households that had at least 1 participant in the International Polar Year Adult Inuit Health Survey for Nunavut. At the time of the survey, the total population of Inuit children aged 3–5 years in the 16 surveyed communities was 1,487, representing 77% of children in this age range in Nunavut. A total of 537 households were contacted, 75 of whom refused participation and 74 cancelled or failed to attend the study appointment. One child per household was selected to participate in the study. For households with 2 children or more in the target age range, the child whose birthday was closest to the date of the survey was selected to be the participant. Thus, 388 children were recruited into the study. Informed consent was obtained from the primary caregiver. The Nunavut Research Institute and the Institutional Review Board of the McGill Faculty of Medicine approved the study.

### Ascertainment of food security and vitamin D status

Members of the research team conducted in-person interviews with primary caregivers in English or Inuit language or dialects. Food security was assessed using the 18-item Household Food Security Survey Module of the United States Department of Agriculture (25), with slight modifications by Indian and Northern Affairs Canada (26). Eight of these 18 questions are specific to food security status of children in the household and 10 are specific to adults. Food security status of the child and adult was determined using definitions established by Health Canada based on the number of affirmative responses on the questionnaire (27) and was divided into food secure, moderately food insecure and severely food insecure. Based on the presumption that adults may go hungry at the expense of providing food for their children, we examined the effect of child and adult food security on respiratory events separately. While both adult and child food insecurity affect children's health, child food insecurity specifically has been associated with greater adverse health effects (28).

Blood was drawn from a subset of children (n=279) in the summer, as not all caregivers consented to the collection of a blood sample on their child. Plasma 25-hydroxyvitamin D3 concentrations, hereafter referred to as vitamin D, were measured using LIAISON total 25(OH)D at McGill University. Detailed methods for vitamin D measurement were reported previously (29). For the purpose of this study, vitamin D status was determined using the Canadian Pediatric Society guidelines (30), with vitamin D deficiency, insufficiency, and sufficiency defined as plasma levels <25 nmol/L, ≥25 and <75 nmol/L, and ≥75 nmol/L, respectively.

### Respiratory outcome measures

The in-person interview included the administration of demographic questionnaires and a questionnaire about respiratory illnesses and symptoms based on the standard ATS-DLD 78c questionnaire (31). The primary outcome consisted of the parental report of an episode of severe chest infection before age 2 years (“Did your child have a severe chest infection before s/he was 2 years old?”), which consisted of a report of bronchiolitis, pneumonia, and asthma. Asthma was included in this definition because it is typically triggered by a viral infection in this preschool age group. As a secondary outcome, we examined the parental report of health centre visits for a respiratory problem in the past 12 months (“In the last 12 months, did you ever have to take your child to the health centre/hospital for a cough, wheezing, or breathing problems?”). The prevalence of respiratory symptoms in this population has been reported previously (32).

### Statistical analysis

We performed a descriptive analysis of general characteristics of the participants and multivariate logistic regression analysis to assess the association between food insecurity and each of the 2 respiratory outcomes. Age and sex were forced as obligatory covariates, while other covariates were included in the final model if they had a p-value of <0.15 on bivariate analysis with the outcome of interest. Covariates considered include: household crowding index [a continuous measure defined as the number of household members divided by the number of rooms in the household, where rooms include bedrooms, kitchen, and living rooms (33)], ever breastfed, birth weight, in utero smoke exposure, presence of a smoker in the house, serum vitamin D level, serum haemoglobin level, serum ferritin level, presence of a hunter in the house, type of housing, whether the household has reported mould or is in need of repairs, and the child's location during the day.

A subgroup analysis was performed among the 279 participants who had a serum vitamin D level available. We performed multivariate logistic regression analysis to assess the association between vitamin D status and each of the 2 respiratory outcomes. Covariates include age, sex, body mass index (BMI) and the covariates included in the respective multivariate models for food security. *p*-Values are 2-sided. All analyses were performed using R, version 3.0.2 (www.r-project.org).

## Results

General characteristics of the 388 children are presented in Table I, with stratification by respiratory outcomes presented in Supplementary Tables I and II. The mean age of this cohort was 3.9 (SD 0.8) years. The majority of these children had been breastfed (64.9%) and had been exposed to tobacco smoke in utero (81.4%) or passively in the house (89.4%). Most households were crowded [crowding is defined as more than 1 person per room (33)], with the median household crowding index at 3.0 people per room (IQR 1.5, 2.5). Summer serum vitamin D levels were available for 279 children, with a median of 48.4 nmol/L (IQR 33.1, 71.8), which is in the insufficient range according to the Canadian Pediatric Society (30).

**Table I:** General characteristics of the study cohort (n = 388)

Age, years, mean (SD)	3.9 (0.8)
Sex, female, n (%)	204 (52.6)
Height (cm), mean (SD)	103.8 (7.2)
Weight (kg), mean (SD)	20.1 (4.1)
Body mass index (BMI), median (IQR)	18.1 (17.1, 19.4)
Birth weight (kg), mean (SD)	2.6 (1.5)
Serum vitamin D performed, n (%)	279 (71.9)
Serum vitamin D level, median (IQR)	48.4 (33.1, 71.8)
Ever breastfed, n (%)	252 (64.9)
Household crowding index, median (IQR)	3.0 (1.5,2.5)
Smokers in the house, n (%)	347 (89.4)
In utero smoke exposure, n (%)	316 (81.4)
Active hunter in the household, n (%)	269 (69.3)
Type of housing, n (%)	
Public	267 (68.8)
Non-public	107 (27.6)
Reported mould or in need of repairs, n (%)	
Mould	20 (5.2)
In need of repairs	87 (22.4)
Both	43 (11.1)
Neither	217 (55.9)
Child's location during the day, n (%)	
Home	238 (61.3)
Day care	129 (33.2)
Homecare	17 (4.4)
Reported severe chest infection before age 2 years, n (%)	112 (28.9)
Reported health centre visit for a respiratory problem in the past 12 months, n (%)	160 (41.2)

Food security data were available for 374 of the 388 participating households. Only a minority of households was categorized as food secure: 41.0% of children, 30.7% of adults and 28.6% of households were food secure. Among the food insecure subgroup, 22.7% of children, 28.8% of adults and 32.5% of households were severely food insecure. A total of 112 (28.9%, data available on 345 children) children had a reported severe chest infection before age 2 years and 160 (41.2%, data available on 378 children) had a reported health centre visit for respiratory problems in the past 12 months.

### Association between food security and respiratory outcomes

Food insecurity was not associated with reported severe chest infections before 2 years, whether it was defined using the child or adult scale (Table II). In multivariate analysis including the child food security scale, household crowding [odds ratio (OR)=1.51, 95% confidence interval (CI) 1.09–2.09, p=0.01] and higher birth weight (OR=1.21 per 1 kg, 95% CI 1.02–1.43, p=0.03) were positively associated with reported severe chest infections before 2 years. These ORs are similar when the adult food security scale was used in the multivariate analysis (Table II). Although the presence of a smoker in the household was associated with reported severe chest infections in the bivariate analysis (p=0.06), given that only 10.6% of households did not have a smoker, this variable was omitted from the multivariate analysis. Furthermore, removing this variable did not significantly change the results of the analysis (data not shown, available upon request).

**Table II:** Effect of child and adult food security on respiratory events

Reported severe chest infection before age 2 years				

	Child food security		Adult food security	
		
	OR (95% CI)	p	OR (95% CI)	p
Age, per year	1.09 (0.81–1.46)	0.56	1.10 (0.82–1.48)	0.52
Sex, female	0.76 (0.47–1.22)	0.26	0.76 (0.47–1.23)	0.26
Household crowding index	1.51 (1.09–2.09)	0.01	1.46 (1.05–2.03)	0.03
Birth weight, per kg	1.21 (1.02–1.43)	0.03	1.22 (1.03–1.45)	0.02
Child score (vs. secure)				
Moderately insecure	0.92 (0.53–1.60)	0.76		
Severely insecure	0.95 (0.51–1.78)	0.88		
Adult score (vs. secure)				
Moderately insecure			1.18 (0.65–2.14)	0.58
Severely insecure			1.17 (0.62–2.20)	0.63
				
Reported health centre visit for a respiratory problem in the past 12 months				

	Child food security		Adult food security	
		
	OR (95% CI)	p	OR (95% CI)	p
				
Age, years	0.66 (0.48–0.91)	0.02	0.68 (0.49–0.94)	0.02
Sex, female	0.70 (0.42–1.16)	0.17	0.72 (0.43–1.20)	0.21
Ever breastfed	0.80 (0.47–1.38)	0.42	0.80 (0.47–1.38)	0.42
Vitamin D	1.04 (0.95–1.14)	0.42	1.03 (0.94–1.13)	0.55
Child score (vs. secure)				
Moderately insecure	1.62 (0.89–2.94)	0.11		
Severely insecure	1.06 (0.56–2.02)	0.86		
Adult score (vs. secure)				
Moderately insecure			1.61 (0.85,3 .04)	0.15
Severely insecure			1.39 (0.71–2.70)	0.34

Food insecurity was not associated with reported health centre visits for respiratory problems in the past 12 months. However, increasing age of the child was associated with decreased odds of reported health centre visits for respiratory problems (OR=0.66, 95% CI 0.48–0.91, p=0.02 for the child food security scale).

### Association between vitamin D and respiratory outcomes

Serum vitamin D levels were available in 279 (71.9%) of participating children. A total of 58 (20.8%) of the children were vitamin D sufficient, 186 (47.9%) were insufficient and 35 (9.0%) were deficient. General characteristics were similar between these groups (Supplementary Table III) and also were similar between those with and without vitamin D levels available (Supplementary Table IV).

Vitamin D status was not associated with reported severe chest infections or health centre visits for respiratory problems (Table III). Similar to the multivariate analysis for food security, birth weight was positively associated with reported severe chest infections (OR=1.31, 95% CI 1.06–1.63, p=0.01). Increasing age was associated with decreased odds of a reported health care visit for respiratory problems in the past 12 months (OR=0.70, 95% CI 0.52–0.96, p=0.03).

**Table III:** Predictors of respiratory events among children with serum vitamin D levels available (n = 279)

Reported severe chest infection before age 2 years		

	OR (95% CI)	p
Age, years	1.02 (0.71–1.46)	0.92
Sex, female	0.72 (0.40–1.30)	0.27
BMI	1.06 (0.93–1.20)	0.39
Household crowding	1.32 (0.89–1.96)	0.17
Birth weight, per kg	1.31 (1.06–1.63)	0.01
Vitamin D		
Insufficiency	0.97 (0.45–2.07)	0.93
Deficiency	1.44 (0.52–3.95)	0.48
		
Reported health centre visit for a respiratory problem in the past 12 months		

	OR (95% CI)	p

Age, years	0.70 (0.52–0.96)	0.03
Sex, female	0.76 (0.46–1.26)	0.29
BMI	1.07 (0.95–1.20)	0.25
Ever breastfed	0.75 (0.43–1.28)	0.29
Vitamin D		
Insufficiency	0.71 (0.38–1.35)	0.30
Deficiency	0.57 (0.23–1.46)	0.25

BMI, body mass index.

## Discussion

Respiratory infections, food security and vitamin D deficiency are highly prevalent problems among Inuit children. Using a large cross-sectional survey of Inuit children aged 3–5 years, a population in which passive smoke exposure and household crowding are highly prevalent, we examined the relationship between these 3 conditions and had 4 key findings. First, we found that household crowding was associated with reported severe chest infections. For each increase of 1 unit in the household crowding index (number of household members divided by the number of rooms in a household), a child had 1.5 times the odds of having had a severe respiratory infection before age 2 years. Second, we were unable to demonstrate a significant association between any past-year food insecurity and adverse respiratory outcomes based upon data of reported severe chest infections before age 2 years or health centre visits for respiratory problems in the past 12 months. Third, higher birth weight was associated with reported severe chest infections, while increasing age was associated with decreased odds of health care visits for respiratory problems. Finally, we did not observe an association between vitamin D insufficiency or deficiency and adverse respiratory outcomes.

In a large cross-sectional survey of 1901 Inuit households, food insecurity was present in 62.6% of households (34). While recent statistics suggest a decrease in food insecurity prevalence in Nunavut (36.2% in 2011–2012), it remains 4 times the Canadian average (35). While many studies suggest that food insecurity among children have adverse effects, including higher rates of chronic illnesses, iron-deficiency anaemia and behavioural problems (36–38), we did not find an association between respiratory infections and food insecurity. Food quality, which is not assessed in most studies, characteristics of the study population as well as methodologies differences may explain the differential findings (39–41). Further studies are needed to clarify the mechanistic pathways between food insecurity, malnutrition and predisposition to infections.

Vitamin D deficiency and insufficiency are highly prevalent problems among Inuit children. In our study population, approximately 80% of children are vitamin D insufficient. This contrasts the prevalence of vitamin D insufficiency (<30 ng/mL or 75 mmol/L) of 37% among children with asthma (42) and 54% among inner-city school-aged children from the United States (43). The high prevalence of vitamin D insufficiency among Inuit children has put into question whether the use of norms for individuals of European descent is appropriate for the Inuit population (44,45), particularly given evidence suggesting that vitamin D may have differential effects on cytokines and hormones in different ethnic populations (46,47) and that ancestry may influence the levels of vitamin D binding proteins (48). Taken together, these studies suggest that the role of vitamin D is likely race/ethnicity-dependant. In this study, we did not find an association between vitamin D levels and respiratory events among Inuit children, despite an overall low median level of vitamin D (48.4 nmol/L). Several observational studies in non-Inuit children have reported an association between vitamin D deficiency and the incidence and severity of respiratory diseases (49,50) and postulated that vitamin D may have immunomodulatory effects, while others did not document an association between lower vitamin D levels and LRTI (51,52). Although the mechanism underlying the interaction between vitamin D and respiratory infections has yet to be elucidated, it would be valuable in explaining the discordant results between studies. Randomized clinical trials suggest that vitamin D supplementation in children may be associated with decreased respiratory infections (24,53) and more trials are on-going. However, the heterogeneity of methodologies in these trials, including the use of different doses, outcomes and populations, warrants cautious interpretation of the results (54). Vitamin D deficiency leads to preventable diseases such as rickets, and supplementation is clearly beneficial in those cases. However, our study results do not demonstrate an association between vitamin D insufficiency or deficiency and reported respiratory infections among Inuit children.

Consistent with our findings, household overcrowding has been associated with lower respiratory infections among Inuit children. In addition to increasing the risk of person-to-person transmission of pathogens, a recent study found that overcrowding was significantly associated with a higher allostatic load, a measure of chronic stress (55). Recently, Ruiz-Castell et al. found that food insecurity was associated with household crowding among school-aged children in the Arctic Quebec, Canada (56). Importantly, household crowding remains a prevalent problem among Canadian Inuit children, with 36.3 and 9.7% living in household with more than 1 and 1.5 person per room, respectively (57). This is compared to 6.7 and 1.6%, respectively, among non-Aboriginal Canadian children (57). While we did not find an association between food insecurity and respiratory infections even in the bivariate analysis, it is possible that household crowding confounded this relationship as household crowding has been associated with food insecurity (56). Overcrowding remains a critical public issue among the Inuit, one that has been consistently associated with health problems in this and other indigenous populations (9,58–60). Interventions are urgently needed to address the overcrowding issue as they may have the greatest health benefits and further studies are warranted to examine the impact of decreasing household crowding on the health of Inuit children.

In this study, we found that higher birth weight was positively associated with respiratory infections in the first 2 years of life. While most studies have focused on low birth weight and respiratory morbidity in infancy, others have found the opposite, specifically that high birth weight was associated with post-bronchiolitis wheezing in infancy (61) and RSV LRTI (62). Although the mechanism remains to be elucidated, it has been suggested that macrosomia may be associated with an altered immunologic phenotype (63). Macrosomia may also be related to maternal diabetes, which may alter the child's immune response through foetal programming (64). We found that increasing age was associated with decreased odds of health care centre visits.

Our study has several strengths. First, this study, which included 388 Inuit children, is one of the largest surveys performed in this population. The survey was performed in 16 Northern communities. Participating children represent 26.1% of all children in the target age range in these communities and 20.1% of all children in the target age range in Nunavut. Second, we assessed child and adult food security separately while most studies examine food security as a household measure. The separate assessment of child and adult food security may provide a better assessment than a single overall measure and sheds light on the effect of both child and adult food status on the child's health.

Several limitations in this study are noteworthy. First, the outcomes assessed in this study, reported severe respiratory infections before age 2 years and health centre visits for respiratory problems in the past year, were assessed in a survey and may be subject to recall bias. However, we purposely chose more severe and clinically significant respiratory outcomes (rather than symptom-based outcomes, which may be even more prone to recall bias) to minimize bias. Second, vitamin D levels were measured on a subgroup of the study cohort only (71.9%), although the characteristics of the subjects who had a vitamin D level available did not differ from those who did not have a vitamin D level available. Third, in examining the relationship between vitamin D and respiratory infections, there may be unmeasured confounding as we did not have data on other important variables, such as the activity level of the children, particularly the amount of time spent outdoors, exposure to sunlight, or the child's vitamin D level during the first 2 years of life. Similarly, there may be unmeasured confounding in the association between household crowding and respiratory infections. Fourth, while this is a large survey, because food insecurity and respiratory infections are so prevalent among Inuit children, it is possible that there was insufficient power to detect an association between the 2 conditions. Finally, food security was assessed over the year prior to the survey, which does not overlap with 1 of the outcomes, which reported severe respiratory infections before age 2 years.

## Conclusion

In a large cohort of Inuit children, we found that child and adult food insecurity, as well as vitamin D deficiency and insufficiency, were not significantly associated with reported severe respiratory infections in the first 2 years of life or visits to a health care centre for respiratory problems in the past 12 months. However, household crowding was significantly associated with reported respiratory infections, which is consistent with previous studies in this population. Further studies are warranted to examine the impact of living conditions improvements, specifically decreasing household crowding, on the respiratory health of Inuit children.

## Supplementary Material

Food insecurity, vitamin D insufficiency and respiratory infections among Inuit children
