# Supplementary material for: Food insecurity, vitamin D insufficiency and respiratory infections among Inuit children
Source: Int J Circumpolar Health. 2016 Feb 15;75:10.3402/ijch.v75.29954. doi: 10.3402/ijch.v75.29954 (PMC4759828; doi:10.3402/ijch.v75.29954)
Supplement: Food insecurity, vitamin D insufficiency and respiratory infections among Inuit children [file IJCH-75-29954-s001.pdf]

Supplemental Table 1. General characteristics of the children with and without reported severe respiratory infection before age 2 years.

	With severe respiratory infection before age 2 years n=112	Without severe respiratory infection before age 2 years n=233
Age, years, mean(SD)	3.9(0.8)	3.9(0.8)
Sex, female, n(%)	53(47.3)	127(54.5)
Height, cm, mean(SD)	103.4(7.3)	104.2(7.2)
Weight, kg, mean(SD)	20.2(4.0)	20.2(4.4)
BMI, median(IQR)	18.2(17.2,19.7)	18.1(17.0,19.3)
Birthweight, kg, mean(SD)	2.8(1.4)	2.5(1.5)
Serum vitamin D level, median(IQR)	42.6(27.0,67.3)	48.1(33.6,71.9)
Ever breastfed, n(%)	72(64.3)	157(67.4)
Household crowding index, median(IQR)	2.0(1.7,2.7)	2.0(1.5,2.4)
Smokers in the house, n(%)	97(86.6)	210(90.1)
In utero smoke exposure, n(%)	92(81.3)	190(81.5)
Type of housing, n(%)		
Public	77(68.8)	157(67.4)
Non-public	33(29.5)	66(28.3)
Reported mold or in need of repair, n(%)		
Mold	3(2.7)	12(5.2)
In need of repairs	23(20.5)	50(21.5)
Both	17(15.2)	22(9.4)
Neither	65(58.0)	134(57.5)
Child's location during the day, n(%)		
Home	70(62.5)	135(57.9)
Daycare	37(33.0)	86(36.9)
Homecare	5(4.5)	12(5.2)

BMI= Body mass index

Supplemental Table 2. General characteristics of the children with and without health center visit for respiratory problem in the past 12 months.

	With health center visit for a respiratory problem in the past 12 months n=160	Without health center visit for a respiratory problem in the past 12 months n=218
Age, years, mean(SD)	3.8(0.8)	3.9(0.8)
Sex, female, n(%)	80(50.0)	120(55.0)
Height, cm, mean(SD)	102.9(7.0)	104.7(7.3)
Weight, kg, mean(SD)	19.9(4.1)	20.4(4.2)
BMI, median(IQR)	18.3(17.0,19.7)	18.0(17.1,19.2)
Birthweight, kg, mean(SD)	2.6(1.5)	2.6(1.5)
Serum vitamin D level, median(IQR)	55.4(33.4,75.7)	45.4(33.0,63.6)
Ever breastfed, n(%)	95(59.4)	153(70.2)
Household crowding index, median(IQR)	2.0(1.5,2.5)	2.0(1.5,2.5)
Smokers in the house, n(%)	143(89.4)	196(89.9)
In utero smoke exposure, n(%)	132(82.5)	178(81.7)
Type of housing, n(%)		
Public	113(70.6)	147(67.4)
Non-public	42(26.3)	64(29.4)
Reported mold or in need of repair, n(%)		
Mold	9(5.6)	11(5.0)
In need of repairs	39(24.4)	48(22.0)
Both	22(13.8)	20(9.2)
Neither	82(51.3)	128(58.7)
Child's location during the day, n(%)		
Home	94(58.8)	140(64.2)
Daycare	57(35.6)	70(32.1)
Homecare	9(5.6)	8(3.7)

BMI= Body mass index

Supplemental Table 3. General characteristics of the children in whom serum vitamin D levels were available, stratified by vitamin D levels. (cutoffs based on the Canadian Pediatric Society recommendations)

	Vitamin D sufficient (>=75 nmol/L) n=58	Vitamin D insufficient (25-75nmol/L) n=186	Vitamin D deficient (<25 nmol/L) n=35
Age, years, mean(SD)	3.7 (0.8)	4.0 (0.8)	4.0 (0.8)
Sex, female, n(%)	35 (60.3)	91 (48.9)	22 (62.9)
Height, cm, mean(SD)	102.0 (7.9)	104.4 (7.2)	104.4 (7.2)
Weight, kg, mean(SD)	19.4 (4.2)	20.1 (3.6)	20.5 (4.5)
BMI, median(IQR)	18.0 (16.8,19.0)	18.1 (17.2,19.4)	18.2 (16.6,19.3)
Birthweight, kg, mean(SD)	2.4 (1.4)	2.5 (1.5)	3.3 (1.5)
Serum vitamin D level, median(IQR)	96.6 (84.1,108.3)	45.8 (34.7,58.5)	19.3 (18.0,23.1)
Ever breastfed, n(%)	34 (58.6)	122 (65.6)	26 (74.3)
Household crowding index, median(IQR)	2.0 (1.5,2.5)	2.0 (1.6,2.5)	2.2 (1.9,2.5)
Smokers in the house, n(%)	53 (94.6)	162 (87.1)	32 (91.4)
In utero smoke exposure, n(%)	55 (94.8)	151 (81.2)	26 (74.3)
Type of housing, n(%)			
Public	43 (74.1)	124 (66.7)	24 (68.6)
Non-public	15 (25.6)	52 (28.0)	9 (25.7)
Reported mold or in need of repair, n(%)			
Mold	2 (3.4)	11 (5.9)	1 (2.9)
In need of repairs	13 (22.4)	42 (22.6)	6 (17.1)
Both	6 (10.3)	21 (11.3)	4 (11.4)
Neither	35 (60.3)	98 (52.7)	22 (62.9)
Child's location during the day, n(%)			
Home	34 (58.6)	115 (61.8)	20 (57.1)
Daycare	20 (34.5)	65 (34.9)	13 (37.1)
Homecare	4 (6.9)	5 (2.7)	1 (2.9)

BMI= Body mass index

Supplemental Table 4. General characteristics of children with and without serum vitamin D levels available.

	Vitamin D available (n=279)	Vitamin D not available (n=109)
Age, years, mean(SD)	3.9 (0.8)	3.8 (0.8)
Sex, female, n(%)	148 (53.0)	56 (51.3)
Height, cm, mean(SD)	104.0 (7.4)	103.5 (6.6)
Weight, kg, mean(SD)	20.0 (3.9)	20.4 (4.7)
BMI, median(IQR)	18.1 (17.0, 19.3)	18.4 (17.1, 19.6)
Birthweight, kg, mean(SD)	2.5 (1.5)	2.6 (1.5)
Ever breastfed, n(%)	182 (65.2)	70 (64.2)
Household crowding index, median(IQR)	2.0 (1.5,2.5)	2.0 (1.5,2.5)
Smokers in the house, n(%)	247 (88.5)	100 (91.7)
In utero smoke exposure, n(%)	232 (83.1)	84 (77.0)
Type of housing, n(%)		
Public	191 (68.5)	76 (69.7)
Non-public	76 (28.3)	31 (28.4)
Reported mold or in need of repair, n(%)		
Mold	14 (5.0)	6 (5.5)
In need of repairs	61 (21.9)	26 (23.9)
Both	31 (11.1)	12 (11.0)
Neither	155 (55.6)	62 (56.9)
Child's location during the day, n(%)		
Home	169 (60.6)	69 (63.3)
Daycare	98 (35.1)	31 (28.4)
Homecare	10 (3.6)	7 (6.4)

BMI= Body mass index
